# YKL-40 correlates with the phenotype of asthma

**DOI:** 10.1186/1939-4551-8-S1-A34

**Published:** 2015-04-08

**Authors:** Krzysztof Specjalski, Ewa Jassem

**Affiliations:** 1 Medical University of Gdansk, Poland

## Background

YKL-40 is a chitinase-like protein synthesized by human macrophages, monocytes, and neutrophils. Although it is not specific, YKL-40 has been shown to correlate with asthma, its severity and level of control. As asthma is heterogeneous, it would be useful to determine whether the marker’s levels correlate with phenotypes of the disease. The aim of the study was to investigate the relevance of YKL-40 as a biomarker of asthma phenotype.

## Methods

Level of YKL-40 was determined by means of immunoassay in sera of 167 asthmatics (116 women, 51 men; aged 18-88; mean age: 49 years) and 81 healthy controls (50 women, 31 men; aged 18-86; mean age: 48 years). On the basis of clinical criteria asthmatics were divided into four groups: atopic – 83 patients, non-atopic - 63, aspirin asthma - 12, asthma with underlying vasculitis- 9. Differences between groups were compared with the use of U-Mann-Whitney’s test. Correlations between variables were assessed with Pearson’s test.

## Results

YKL-40 levels were significantly higher, on average, in asthmatics compared to control group (mean levels: 66,8 U/l and 44,9 U/l respectively; p<0,001). YKL-40 correlated with lack of asthma control (p<0,001) and diagnosis of exacerbation (p<0,001). The highest mean concentration was found in atopic asthmatics – 72U/l which was significantly higher compared to non-atopic patients – 61 U/l (p<0,05). Weak correlations were found between YKL-40 levels and CRP as well as FEV_1_. However no correlations have been found between YKL-40 level and sex, blood eosinophils count and neutrophils count.

## Conclusions

YKL-40 correlates with asthma control, atopy, FEV_1_ and CRP. The latter suggests that YKL-40 concentration may depend on severity of inflammation what seems confirmed by the elevated levels in numerous inflammatory diseases and neoplasms.

